# Refractory rhino-orbito-cerebral mucormycosis treated with intraconal amphotericin B

**DOI:** 10.5935/0004-2749.20220009

**Published:** 2022

**Authors:** Juliana A de Guimarães, Frederico C Moura

**Affiliations:** 1 Department of Ophthalmology and Otorhinolaryngology, Universidade Estadual de Campinas, Campinas, SP, Brazil; 2 Neuro-Ophthalmology Service, Department of Ophthalmology and Otorhinolaryngology, Universidade Estadual de Campinas, Campinas, SP, Brazil; 3 Universidade de São Paulo, São Paulo, SP, Brazil

**Keywords:** Mucormycosis, Orbital cellulitis, Amphotericin B, Orbit, Fungal infections, Mucormicose, Celulite orbitária, Anfotericina B, Órbita, Infecções fúngicas

## Abstract

We report the case of a 46-year-old diabetic man receiving treatment for rhino-orbital-cerebral mucormycosis with liposomal amphotericin B and surgical debridement. The patient’s condition worsened clinically, accompanied by the loss of ocular motility and a visual acuity of absence of light perception. Radiological extension of the infection was evidenced, with invasion of the cavernous sinus. Based on ophthalmological findings, exenteration (a potentially disfiguring procedure) was indicated, but we opted for wide surgical debridement and administration of amphotericin B via intraconal catheter. Clinical improvement and resolution of inflammation occurred after 2 weeks of treatment. Thus, rhino-orbital-cerebral mucormycosis was effectively controlled through intraconal administration of amphotericin B, while avoiding exenteration. The intervention should be considered as an adjuvant treatment in selected rhino-orbital-cerebral mucormycosis cases before attempting exenteration.

## Abstract (pt)

Os autores relatam o caso de um homem diabético de 46 anos de idade em tratamento para mucormicose rino-orbital-cerebral com anfotericina B lipossomal e desbridamento cirúrgico. Piora clínica foi observada, com acometimento de motilidade ocular e acuidade visual de ausência de percepção luminosa, sendo evidenciada extensão radiológica da infecção, com invasão do seio cavernoso. Com base nos achados oftalmológicos, uma exenteração, procedimento potencialmente desfigurante, foi indicada, mas optamos por realizar amplo desbridamento cirúrgico e administração intraconal de anfotericina B por cateter. Melhora clínica e resolução da inflamação ocorreram após duas semanas de tratamento. Dessa forma, a mucormicose rino-orbital-cerebral foi efetivamente controlada com a administração intraconal de anfotericina B, evitando a exenteração. A intervenção deve ser considerada como um tratamento adjuvante em casos selecionados de mucormicose rino-orbital-cerebral antes que a exenteração seja realizada.

## INTRODUCTION

Mucormycosis is a severe and often fatal acute fungal disease. The angio-invasive behavior of the fungus leads to thrombosis, tissue necrosis, and invasion of adjacent tissues, thereby minimizing the local availability of systemic antifungals and making aggressive surgical approaches necessary^(1)^. When affecting the sinus, rhinoorbito-cerebral mucormycosis (ROCM) can extend to the orbital tissues, increasing the risk of central nervous system (CNS) involvement and death. In such cases, exenteration is commonly indicated^(2)^, although no consensus exists regarding the timing of this potentially disfiguring and traumatic procedures^(3,4)^. Several alternatives have been proposed for this purpose, one of which is intraconal irrigation with antifungals^(3)^.

Through this report, we have described a case of refractory ROCM that was successfully treated with amphotericin B via intraconal catheter, avoiding exenteration. The study complied with the guidelines of the Declaration of Helsinki.

## CASE REPORT

A 46-year-old man with a history of poorly controlled type 2 diabetes presented with pain, hyperemia, and proptosis of the right eye (OD) associated with right peripheral facial paralysis and purulent nasal discharge for 3 weeks. Upon clinical examination, proptosis and hyperemia were observed in OD, but extrinsic ocular motility and pupillary reflexes were normal in both eyes (OU). Right peripheral facial palsy was evidenced, but, on fundoscopy, no edema or atrophy of the optic nerve was noticed in either eye. No baseline visual acuity (VA) was recorded.

**Figure 1:**
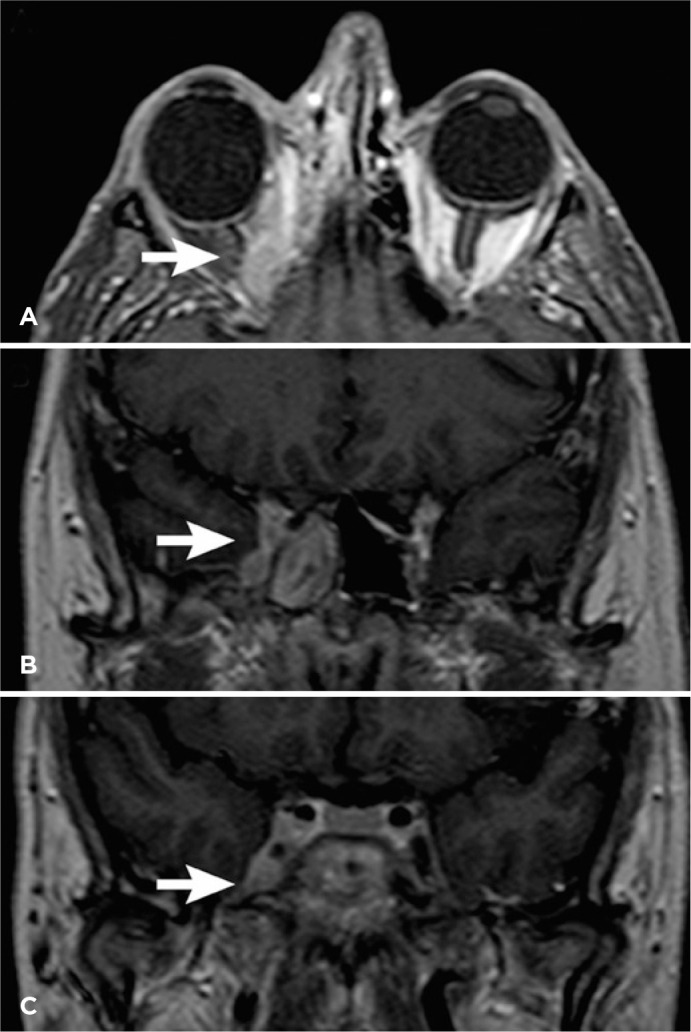
Nuclear magnetic resonance. (A) Axial section, showing an upper orbital lesion affecting the right orbital apex (arrow). (B) Coronal section, showing veiling of the sphenoid sinus on the right (arrow). (C) Coronal section, showing involvement of the cavernous sinus on the right (arrow).

**Figure 2:**
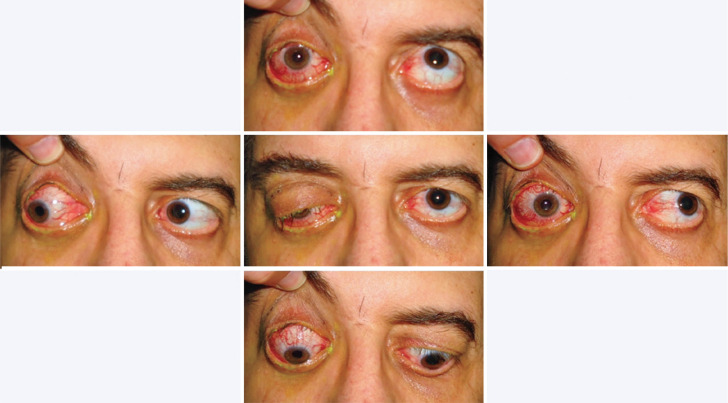
Images showing ptosis and deficits in ocular motility in the right eye, mainly when attempting to elevate and adduct.

CT scans of the skull and orbits revealed a superior nasal orbital lesion associated with veiling of the maxillary and ethmoid sinuses on the right side. Diagnostic sinusectomy revealed fungal hyphae compatible with mucormycosis, and the patient was started on intravenous amphotericin B deoxycholate. After a month’s time, ophthalmoplegia developed and progression to the cavernous sinus was visible on the orbital MRI (Figure 1). Amphotericin B deoxycholate was replaced with liposomal amphotericin B owing to nephrotoxicity and clinical-radiological progression of the condition. Despite the intravenous treatment and 3 sinusectomies performed by the otorhinolaryngology team, orbital proptosis increased and ophthalmoplegia worsened, which resulted in severe visual impairment. At this point of time, VA was absence of light perception in OD and 20/20 in the left eye, with restricted ocular motility (Figure 2) and an absolute afferent pupillary defect in OD. The only finding on biomicroscopy was conjunctival hyperemia in OD. Intraocular pressure was 12 mmHg in OU, and the optic nerve in OD displayed atrophy on fundoscopy.

**Figure 3:**
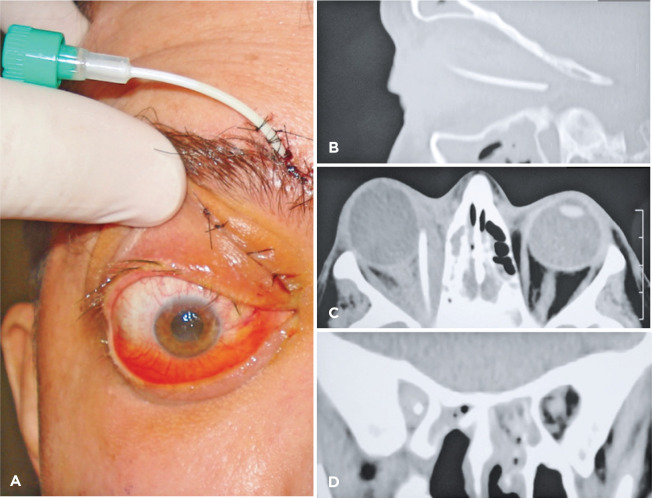
(A) Image showing the upper nasal positioning of the catheter. (B, C, and D) Computed tomography of orbits showing the intraconal positioning of the catheter (arrows). (B) Sagittal section. (C) Coronal section. (D) Axial section.

**Figure 4:**
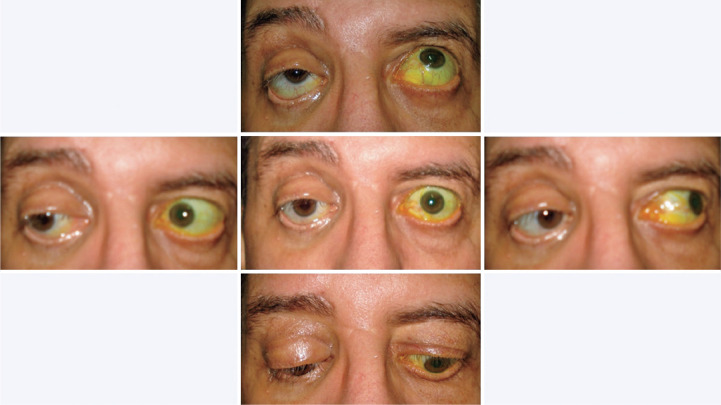
Post-treatment images showing ptosis in the right eye, pseudo-retraction in the left eye, and slight hypotropy and deficit of elevation in the right eye.

Despite the indication for exenteration, we opted for wide debridement of the orbital necrotic tissues and placement of a 16-G-radiopaque polyurethane catheter (Smiths Medical ASD, Minneapolis, MN, USA) in the intraconal region for local administration of amphotericin B deoxycholate (Figure 3). Eleven applications of an average of 1-mL amphotericin B at the dosage 3 mg/mL were performed. The only adverse effect was self-limited, mild chemosis. After 2 weeks of treatment, extrinsic ocular motility improved and inflammation resolved (Figure 4). The patient was discharged 10 days after the last intraconal amphotericin B application, but his vision in OD did not recover. After 2 months of outpatient follow-up without any specific treatment for mucormycosis, the patient remained without complaints.

## DISCUSSION

Mucormycosis is caused by fungi belonging to the order *Mucorales* (class: *Zygomycetes*), with *Rhizopus spp.* as the most commonly associated genus. It is a severe acute disease, with a mortality rate of 46%. The most frequently associated condition is diabetes mellitus, followed by hematological neoplasms, hematopoietic stem cell transplantation, and solid neoplasms. ROCM is the most frequent presentation of mucormycosis, corresponding to 34-58% of all cases^(1)^.

Fungal spores inhaled from the environment trigger sinusitis in predisposed individuals. After invading the blood vessel walls, the fungus causes thrombosis, tissue necrosis, and damage to the adjacent tissues, limiting the local availability of antifungal agents^(3,5)^. The standard management of ROCM is based on the control of predisposing clinical conditions, the intravenous administration of antifungals, and the surgical debridement of the infected tissues^(1)^.

In the recent years, with the purpose of reducing mortality and the need for potentially disfiguring procedures such as exenteration, several alternative, and/or adjuvant treatments have been proposed, including modern antifungal medications (e.g., posaconazole), local antifungals, hyperbaric therapy, and cautious debridement with or without intraoperative freezing biopsy^(3,5)^.

The use of local antifungal agents to treat ROCM was first described by Fleckner and Goldstein in 1969 based on 2 cases treated with amphotericin B deoxycholate intravenously and through topical sinus irrigation^(3)^. Past studies have since then reported the treatment of ROCM with amphotericin B using retrobulbar injections^(4,5)^, surgical packing of the orbit with soaked gauze^(3,6)^, and orbital irrigation through a catheter^(3,7-9)^. Exenteration was necessary in only one of the 14 patients reported to have been treated with local amphotericin B^(3,5-7,9)^. The use of local amphotericin B helps maintain a higher drug tissue concentration. This is important since diffusion to the tissues from the bloodstream is compromised by the presence of necrotic tissues and by the high molecular weight and the strong protein affinity of the amphotericin B molecule^(5)^.

Although exenteration is associated with longer survival in patients with fever^^2^^ and is commonly indicated in the presence of refractory orbital impairment or threat of invasion of the CNS, no clear consensus exists regarding the timing of the procedure^(5)^. Safi et al.^(4)^ reported a case of ROCM associated with left temporal cerebritis on nuclear MRI that was successfully treated with retrobulbar applications of amphotericin B, which resulted in avoidance of exenteration^(4)^. Our patient presented with clinical and radiological evidence of cavernous sinus involvement, making it, to the best of our knowledge, the second case of ROCM associated with CNS involvement that was successfully treated with topical amphotericin B application.

Although it is well-documented, intraorbital amphotericin B irrigation via catheter remains an off-label technique, with no randomized clinical trials defining the ideal dosage and catheter material or the associated risks^(3,6,7)^. In the authors’ opinion, amphotericin B irrigation via catheter offers an advantage over retrobulbar injections due to the lower morbidity resulting from not having to administer a new injection for each dose of amphotericin B administered.

Past *in vitro* studies have suggested that amphotericin B may have a potential for local toxicity^(5)^. This aspect would explain the occurrence of chemosis in the present case as well as the occurrence of chemosis with or without discrete proptosis after treatment onset in other cases reported in the literature^(4,5)^. The inflammatory reaction following the administration of amphotericin B may be reduced by adjusting the dosage and by replacing amphotericin B with liposomal formulations associated with weaker inflammatory response^(10)^. However, despite the associated local toxicity, the adverse effects of amphotericin B reported so far have been mild and self-limiting and they are far outweighed by the therapeutic benefits of the drug used in the treatment of ROCM.
